# Switch-2 determines Mg^2+^ADP-release kinetics and fine-tunes the duty ratio of *Dictyostelium* class-1 myosins

**DOI:** 10.3389/fphys.2024.1393952

**Published:** 2024-06-03

**Authors:** Ralph P. Diensthuber, Falk K. Hartmann, Daniela Kathmann, Peter Franz, Georgios Tsiavaliaris

**Affiliations:** Institute for Biophysical Chemistry, OE 4350, Hannover Medical School, Hannover, Germany

**Keywords:** myosin, myosin-1, actin, duty ratio, kinetics

## Abstract

Though myosins share a structurally conserved motor domain, single amino acid variations of active site elements, including the P-loop, switch-1 and switch-2, which act as nucleotide sensors, can substantially determine the kinetic signature of a myosin, *i.e*., to either perform fast movement or enable long-range transport and tension generation. Switch-2 essentially contributes to the ATP hydrolysis reaction and determines product release. With few exceptions, class-1 myosin harbor a tyrosine in the switch-2 consensus sequence DIYGFE, at a position where class-2 myosins and a selection of myosins from other classes have a substitution. Here, we addressed the role of the tyrosine in switch-2 of class-1 myosins as potential determinant of the duty ratio. We generated constitutively active motor domain constructs of two class-1 myosins from the social amoeba *Dictyostelium discoideum*, namely, Myo1E, a high duty ratio myosin and Myo1B, a low duty ratio myosin. In Myo1E we introduced mutation Y388F and in Myo1B mutation F387Y. The detailed functional characterization by steady-state and transient kinetic experiments, combined with *in vitro* motility and landing assays revealed an almost reciprocal relationship of a number of critical kinetic parameters and equilibrium constants between wild-type and mutants that dictate the lifetime of the strongly actin-attached states of myosin. The Y-to-F mutation increased the duty ratio of Moy1B by almost one order of magnitude, while the introduction of the phenylalanine in switch-2 of Myo1E transformed the myosin into a low duty ratio motor. These data together with structural considerations propose a role of switch-2 in fine-tuning ADP release through a mechanism, where the class-specific tyrosine together with surrounding residues contributes to the coordination of Mg^2+^ and ADP. Our results highlight the importance of conserved switch-2 residues in class-1 myosins for efficient chemo-mechanical coupling, revealing that switch-2 is important to adjust the duty ratio of the amoeboid class-1 myosins for performing movement, transport or gating functions.

## 1 Introduction

Class-1 myosins act primarily at the interface between the actin cytoskeleton and membrane systems, where they provide the mechanical forces and tension to drive membrane rearrangements, coordinate actin remodeling, and accomplish intracellular transport functions (McConnell and Tyska, 2010; McIntosh and Ostap, 2016; Barger et al., 2019; Manenschijn et al., 2019; Lutton et al., 2023). Apart from a modular tail that contains domains and motifs for cargo binding and membrane association (Adams and Pollard, 1989), the class-1 myosins share a globular head through which they interact with actin and hydrolyze ATP to perform fast movement, transport or gating functions (Mezgueldi et al., 2002; Laakso et al., 2008; Lin et al., 2011). A critical parameter of the myosin kinetic cycle is the duty ratio, which defines the fraction of time a myosin spends in the strongly attached actin states relative to its total ATPase cycle time. Low duty ratio myosins are generally fast movers (Johnson et al., 2019) due to a fast release of the hydrolysis products and short-lived strongly-actin bound states (Bloemink and Geeves, 2011), whereas high duty ratio myosins are characterized by a rate limiting ADP release, which prolongs the occupation of states of strong actin interactions (De La Cruz et al., 1999; Amrute-Nayak et al., 2019) and which is considered as prerequisite for the generation of tension and processive movement (Armstrong et al., 2012).

The myosin motor domain contains surface exposed actin-binding loops and harbors in the inner core a conserved nucleotide-binding pocket formed by three structural motifs, termed P-loop, switch-1, and switch-2 that act as nucleotide sensors (Wittinghofer and Geeves, 2016). Their reversible switching between open and closed conformations enables ATP hydrolysis and couples actin-cleft closure to the bending of the relay helix followed by a rotational rigid-body movement of the converter domain that drives the power-stroke concomitant with product release (Preller and Holmes, 2013; Franz et al., 2021). Mutagenic studies have shown that conserved residues within switch-2 are critical for efficient ATP hydrolysis and activation of ATPase activity (Furch et al., 1999; Málnási-Csizmadia et al., 2005; Nagy et al., 2010). Through hydrogen bond formation and Mg^2+^-coordination, switch-2 serves as important regulator of ATP-hydrolysis and product release. A tyrosine residue is found in the switch-2 consensus sequence DIYGFE of various myosins at a position were fast skeletal muscle myosin-2 and a selection of myosins from other classes have an alanine, serine or phenylalanine (Odronitz and Kollmar 2007). Recently, we have shown that physiological changes in the concentration of free Mg^2+^-ions can modulate the kinetic and motor properties of the high duty ratio amoeboid class-1 myosins Myo1E and Myo1D, which contain a tyrosine residue at this position (Fujita-Becker et al., 2005; Dürrwang et al., 2006), but not of the low duty ratio Myo1B with a phenylalanine substitution (Tsiavaliaris et al., 2008). To dissect a potential contribution of this variant residue on the duty ratio of the myosins, we generated mutant constructs, in which the amino acid was replaced by a tyrosine (construct Myo1B^F387Y^) or a phenylalanine (construct Myo1E^Y388F^). In the case of Myo1B, the F387Y mutation sensitized the myosin to modulate its kinetics by free Mg^2+^-ions. The F-to-Y substitution decelerated ADP release and increased the ADP affinity of the actin bound states, affecting duty ratio and motor activity. For Myo1E the Y-to-F substitution produced the opposite effects resulting in loss of Mg^2+^-sensitivity and low duty ratio. Structural models propose a role of this tyrosine residue in mediating interactions between switch-2, Mg^2+^, and the nucleotide important to fine-tune Mg^2+^ADP release and consequently the duty ratio of the myosins.

## 2 Materials and methods

### 2.1 Reagents

Standard chemicals, anti-His antibody, and TRITC-phalloidin were purchased from Sigma; restriction enzymes, polymerases and DNA-modifying enzymes were purchased from MBI-Fermentas and Roche Applied Sciences. The 2’-(3’-)-O-(N′-Methylanthraniloyl) derivatives of ATP (mantATP) and ADP (mantADP) were purchased from Jena Bioscience.

### 2.2 Plasmid construction and protein purification

Expression plasmids pDXA-MyoB-S332E-F387Y-2R and pDXA-MyoE-S336E-Y388F-2R, which encode the constitutively active motor domain constructs of *Dd* myosion-1B and *Dd* myosion-1E harboring mutations F387Y and Y388F, respectively, fused to an artificial lever arm (2R) and a C-terminal His_8_-tag, were generated by PCR using mutagenesis primers 5′-GAT​TTC​AAA​ACC​ATA​AAT​ATC​TAA​AAT​ACC-3′ for introducing mutation F387Y in the motor domain of myosin-1B and 5′-CTC​AAA​ACC​AAA​GAT​ATC​AAG-3′ for introducing mutation Y388F in the motor domain of myosin-1E. *E. coli* strain XL1Blue (Stratagene, Heidelberg) was used for amplification of the plasmids. Myosin constructs were confirmed by sequencing. Wild-type and mutant constructs were produced in *Dictyostelium discoideum* and purified as described (Fujita-Becker et al., 2005; Dürrwang et al., 2006; Tsiavaliaris et al., 2008). Chicken skeletal actin was purified as described (Pardee and Aspudich, 1982). Pyrene-labeled actin was prepared from skeletal actin as described (Criddle et al., 1985).

### 2.3 Steady-state and transient kinetic experiments, *in vitro* motility and landing assays

Steady-state ATPase activity measurements were performed at 25°C in buffer containing 25 mM 2-(4-(2-Hydroxyethyl)-1-piperazinyl)-ethansulfonsäure (HEPES) pH = 7.3, 25 mM KCl, 5 mM MgCl_2_, 1 mM Dithiothreitol and 1 mM ATP using the NADH-coupled assay (Diensthuber et al., 2015). Unless otherwise stated, transient kinetic measurements were performed at 20°C in experimental buffer containing 20 mM 3-(N-morpholino)propanesulfonic acid (MOPS) pH = 7.0, 100 mM KCl, 5 mM MgCl_2_ and 1 mM DTT using a Hi-tech Scientific SF-61DX double-mixing stopped-flow system (TgK Scientific Limited, Bradford on Avon, U.K.). Data were analyzed according to the procedures and kinetic models described (Bagshaw et al., 1974; Millar and Geeves, 1983; Cremo and Geeves, 1998; Batra et al., 1999; Furch et al., 1999). Data for wild-type myosins were depicted from Dürrwang et al., 2006; Tsiavaliaris et al., 2008, respectively, and are listed in the tables as assigned, if not otherwise stated. *In vitro* motility assays were performed at 30 °C using an Olympus IX81 inverted fluorescence microscope as described (Taft et al., 2008). Penta∙His Antibody (Qiagen) was used for the specific attachment of wild-type and mutant myosin constructs on nitrocellulose coated coverslips. Actin filament tracking was performed using DiaTrack 3.05 software. Average actin sliding velocities were obtained from Gaussian fits to the velocity distributions using Origin 2022b software (OriginLab, Northampton, MA, USA). Landing assays were performed as described (Taft et al., 2008).

### 2.4 Molecular dynamics simulations and homology modelling

Mutation Y388F was introduced in the X-ray crystal structure of the Myo1E motor domain (pdb: 1LKX) using the Schrödinger Suite (Schrödinger Inc.), selecting the rotamers with the lowest sterical hindrance, and energy-minimization of the entire model was performed with MacroModel (Schrödinger Inc.), CNSsolve 1.2 (Brunger, 2007), and the OPLS3 force field. Molecular dynamics simulation of Myo1E^Y388F^ was carried out with Gromacs 4.0 (Hess et al., 2008) and OPLS (Optimized Potentials for Liquid Simulations) all-atom force field. The myosin was solvated with the TIP3P explicit water model and neutralized by addition of sodium counter ions as described (Preller et al., 2011). MD simulations were performed in a NpT ensemble (300 K, 1 bar) using Berendsen temperature coupling and Parrinello-Rahman pressure coupling. The particle-mesh Ewald method (Darden et al., 1993) was used for long-range electrostatic interactions. Short-range van der Waals and coulomb forces were treated with 12 Å cutoffs. A 2 fs time step was used during the production runs, and all bond lengths were constrained with the LINCS algorithm (Hess et al., 1997). The coordinates were optimized with the conjugate gradient algorithm to a final force of <10 kJ·mol^-1^·nm^-1^ after energy minimization with the steepest descent algorithm to a force of 1,000 kJ·mol^-1^·nm^-1^. The solvent molecules were equilibrated for 100 ps. A 4 ns equilibration of the entire system was performed to reach a plateau of the root mean square deviation of the backbone atoms. Production runs were performed for 100 ns? Myo1B homology models were generated with Modeller (Šali and Blundell, 1993) using the crystal structure of Myo1E motor domain (pdb: 1LKX) as template. Best models were selected according to the Modeller objective function and the discrete optimized protein energy score (DOPE). Images were generated with Pymol (DeLano scientific).

### 2.5 Equations

$$L\left(\rho \right)=Z∙{\left(1-e^{-\frac{\rho }{\rho_{0}}}\right)}^{n}$$ (1)

$$duty\;ratio=\frac{\tau_{\mathrm{strong}}}{\tau_{\mathrm{total}}}\approx \frac{\frac{1}{k_{-\text{AD}}}}{\frac{1}{k_{\mathrm{cat}}}}$$ (2)

## 3 Results and discussion

### 3.1 Switch-2 mutations do not impair steady-state ATP turnover

**FIGURE 1:**
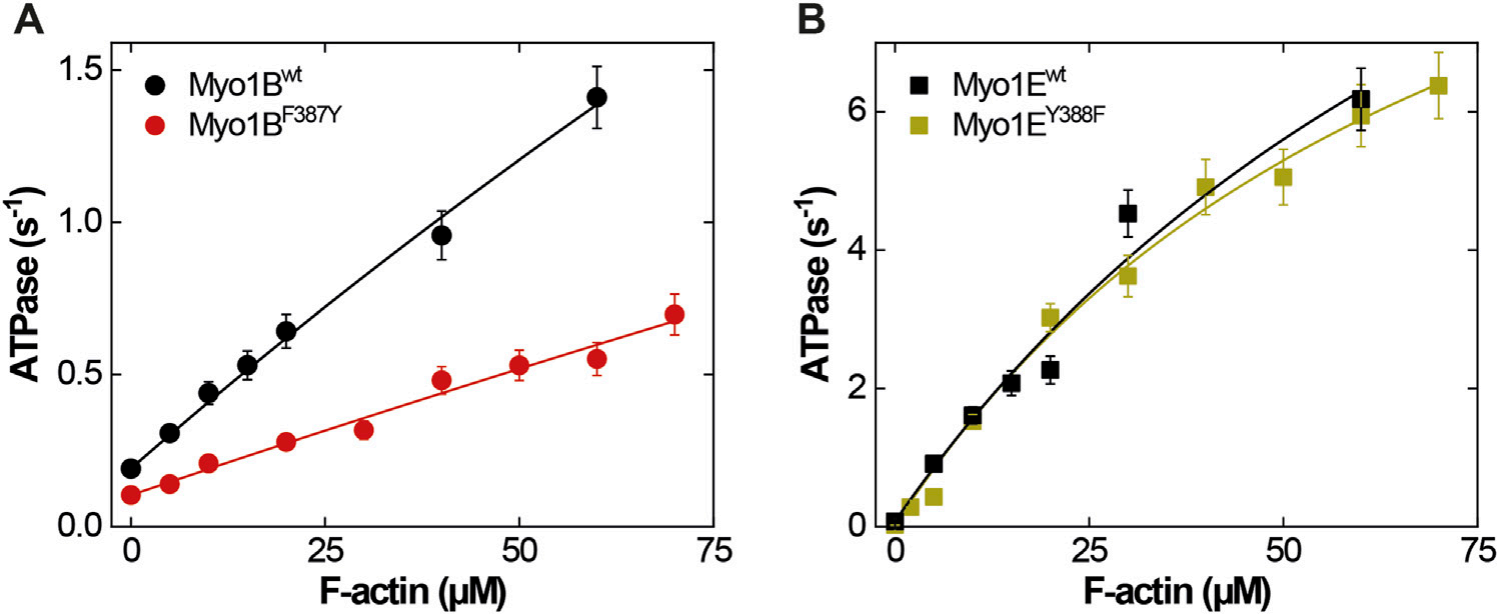
*Steady-state* ATPase activities of wild-type and mutant myosins. **(A)** Actin-activated ATPase of Myo1B^wt^ (black circles) and Myo1B^F387Y^ (red circles). **(B)** Actin-activated ATPase of Myo1E^wt^ (black squares) and Myo1B^F387Y^ (green squares). Michaelis-Menten parameters are summarized in Table 1.

The ATPase activities of the wild-type myosin motor domain constructs Myo1B^wt^ and Myo1E^wt^ and the corresponding switch-2 mutants Myo1B^F387Y^, Myo1E^Y388F^, all carrying the S-to-E mutation at the TEDS site, which mimics the phosphorylation state and transforms the myosins into active, ATPase competent motors (Fujita-Becker et al., 2005; Dürrwang et al., 2006) were examined in the absence and presence of F-actin. Data for wild-type myosins were depicted from (Dürrwang et al., 2006; Tsiavaliaris et al., 2008) if not otherwise stated. Hyperbolic fit of the actin-dependence of the rate of ATP turnover according to Michaelis-Menten (Figure 1) yielded the basal ATPase activity (*k*_basal_), the apparent equilibrium constant of half-maximal activation of maximum ATP turnover (**K**_**app**_), and actin-activated ATPase activity for saturating actin concentrations (***k***_**cat**_). The steady-state parameters are summarized in Table 1. The mutants displayed reduced *k*_basal_ rates compared to the wild-types, while **K**_**app**_ remained largely unaffected. In the case of Myo1B^F387Y^, the actin-activated ATPase activity increased almost linear, which allows only rough estimates of the apparent equilibrium constant (**K**_**app**_) and the maximum ATP turnover rate (***k***_**cat**_). Myo1B^F387Y^ shows a 3-fold reduction in both, ***k***_**cat**_ and the catalytic efficiency ***k***_**cat**_/**K**_**app**_. Latter is a measure of how effectively actin activates the ATPase reaction as defined by the 2^nd^ order rate constant of actin binding in the presence of ATP. These parameters were unaffected in Myo1E^Y388F^. Since we observed different impact of the mutations on the catalytic activity of the myosins, we extended the analysis to transient kinetic experiments and focused our investigations on those steps of the ATPase cycle that determine the occupation and lifetime of the strongly vs. weakly actin-bound states of the myosins.

**TABLE 1:** Actin-activated ATPase activities.

		Myo1B^wt^ ^a^	Myo1B^F387Y^	Myo1E^wt^ ^a^	Myo1E^Y388F^
**Basale ATPase**	*k*_basal_ (s^-1^)	0.16 ± 0.02	0.08 ± 0.01	0.08 ± 0.01	0.02 ± 0.01
**Michaelis-Menten parameters**	***k***_**cat**_ (s^-1^)	3.1 ± 0.4	>1	15.4 ± 3	13.4 ± 2
	**K**_**app**_ (μM)	96 ± 20	>100	91 ± 30	77 ± 17
	***k***_**cat**_/**K**_**app**_ (μM^-1^s^-1^)	0.032 ± 0.008	>0.01	0.17 ± 0.06	0.17 ± 0.05

^a^ Depicted from Dürrwang et al., 2006 or Tsiavaliaris et al., 2008.

### 3.2 Switch-2 mutations produce inverse effects on ADP dissociation, actin affinity, and weak-to-strong actin interactions

**FIGURE 2:**
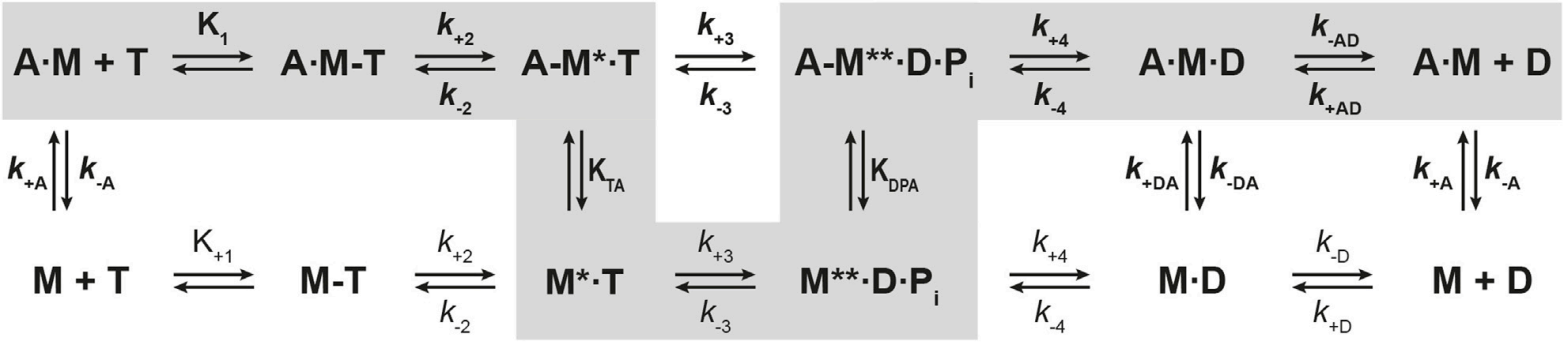
Kinetic scheme of the actomyosin ATPase cycle. Shown are the biochemical pathways of myosin during the interaction with nucleotides in the presence of actin (upper pathway) and absence of actin (lower pathway). Highlighted in grey is the force generating pathway. The biochemical states are defined in terms of the interacting proteins and nucleotides, where A = actin, M = myosin, T = ATP, D = ADP, P_i_ = phosphate. Equilibrium constants and rate constants associated with actin are shown in bold (**K**, ***k***), all others in regular fonts (K, *k*). In the force producing pathway, strongly actin-bound states of myosin are assigned as A·M, A·M-T, A·M·D, weakly actin-bound states as A-M*·T, A-M**·D·P_i_, and actin-detached states as M, M-T, M*·T and M**·D·P_i_. A dash defines a weak interaction, a dot a strong interaction. States assigned with asterisk (*) are fluorescence sensitive conformational transition and isomerisation states. ***k***_**-AD**_ = rate constant of ADP dissociation from actomyosin; ***k***_**+AD**_ = rate constant of ADP binding to actomyosin; *k*_-D_ = rate constant of ADP release from myosin; *k*_+D_ = rate constant of ADP binding to myosin; ***k***_**+A**_ = rate constant of actin binding to myosin; ***k***_**-A**_ = rate constant of actin dissociation from myosin. **K**_**TA**_: equilibrium constant of the actin interaction of myosin with bound ATP. **K**_**DPA**_: equilibrium constant of the actin interaction of myosin in the ADP-P_i_ state. **K**_**A**_: equilibrium constant of actin binding to myosin. **K**_**AD**_: equilibrium constant of actin binding to myosin with bound ADP.

**FIGURE 3:**
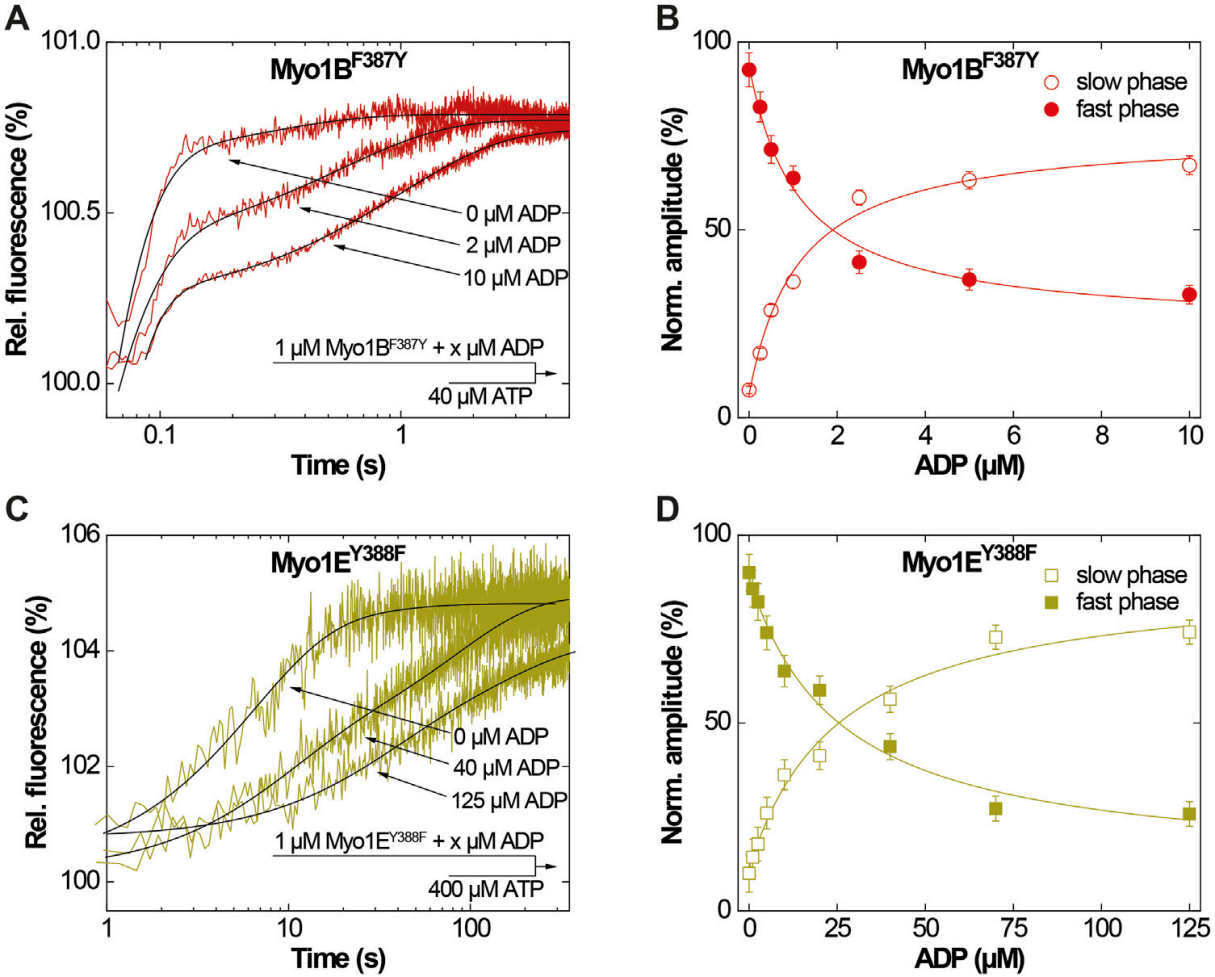
ADP-interactions of Myo1B^F387Y^ and Myo1E^Y388F^ in the absence of actin. **(A)** Single fluorescence transients as monitored upon mixing 1 µM Myo1B^F387Y^ pre-incubated with 0, 2 or 10 µM ADP with excess ATP. **(B)** Normalized amplitudes of the slow phase (open circles) and fast phase (filled circles) as obtained from biexponential fits to the transients observed upon mixing 1 µM Myo1B^F387Y^ pre-incubated with increasing concentrations ADP with excess ATP. **(C)** Single fluorescence transients as monitored upon mixing 1 µM Myo1E^Y388F^ pre-incubated with 0, 40 or 125 µM ADP with excess ATP. **(D)** Normalized amplitudes of the slow phase (open circles) and fast phase (filled circles) as obtained from biexponential fits to the transients observed upon mixing 1 µM Myo1E^Y388F^ pre-incubated with increasing concentrations ADP with excess ATP.

**FIGURE 4:**
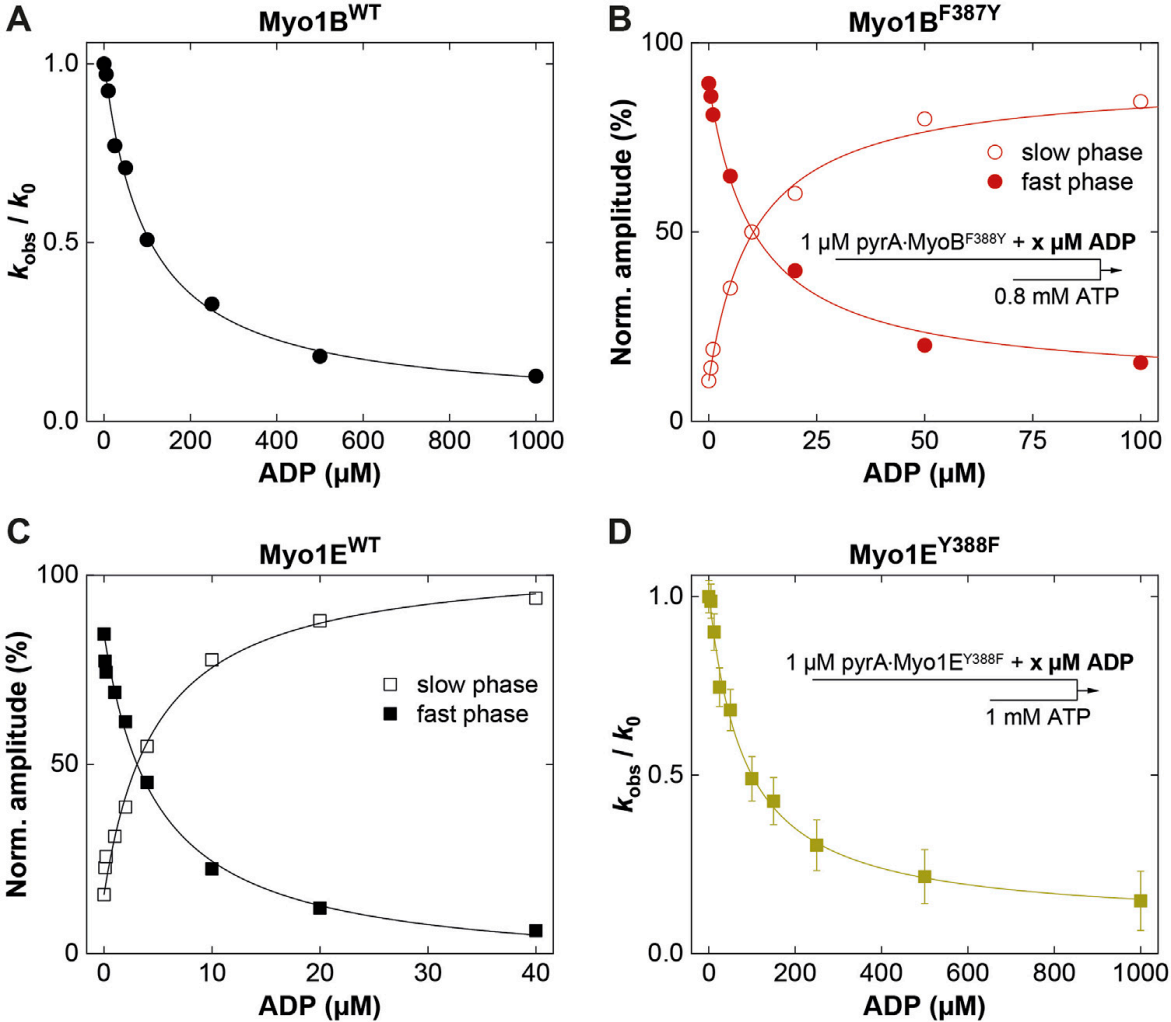
ADP-interactions of wild-type and mutant myosins in the presence of actin. **(A)** Plot of the relative change of the observed rate constant (*k*_obs_/*k*_0_) as obtained from single exponential fits to the fluorescence transients observed upon mixing pyrA∙Myo1B^wt^ in the presence of increasing ADP concentrations with excess ATP. **(B)** Normalized amplitudes of the slow phase (open circles) and fast phase (filled circles) as obtained from biexponentional fits to the fluorescence transients observed upon mixing 1 µM pyrA∙Myo1B^F387Y^ in the presence of increasing ADP concentrations with excess ATP. **(C)** Normalized amplitudes of the slow phase (open squares) and fast phase (filled squares) as obtained from biexponentional fits to the fluorescence transients observed upon mixing pyrA∙Myo1E^wt^ in the presence of increasing ADP concentrations with excess ATP. **(D)** Plot of the relative change of the observed rate constant (*k*_obs_/*k*_0_) as obtained from single exponential fits to the fluorescence transients observed upon mixing 1 µM pyrA∙Myo1E^F388Y^ in the presence of increasing ADP concentrations with excess ATP.

**FIGURE 5:**
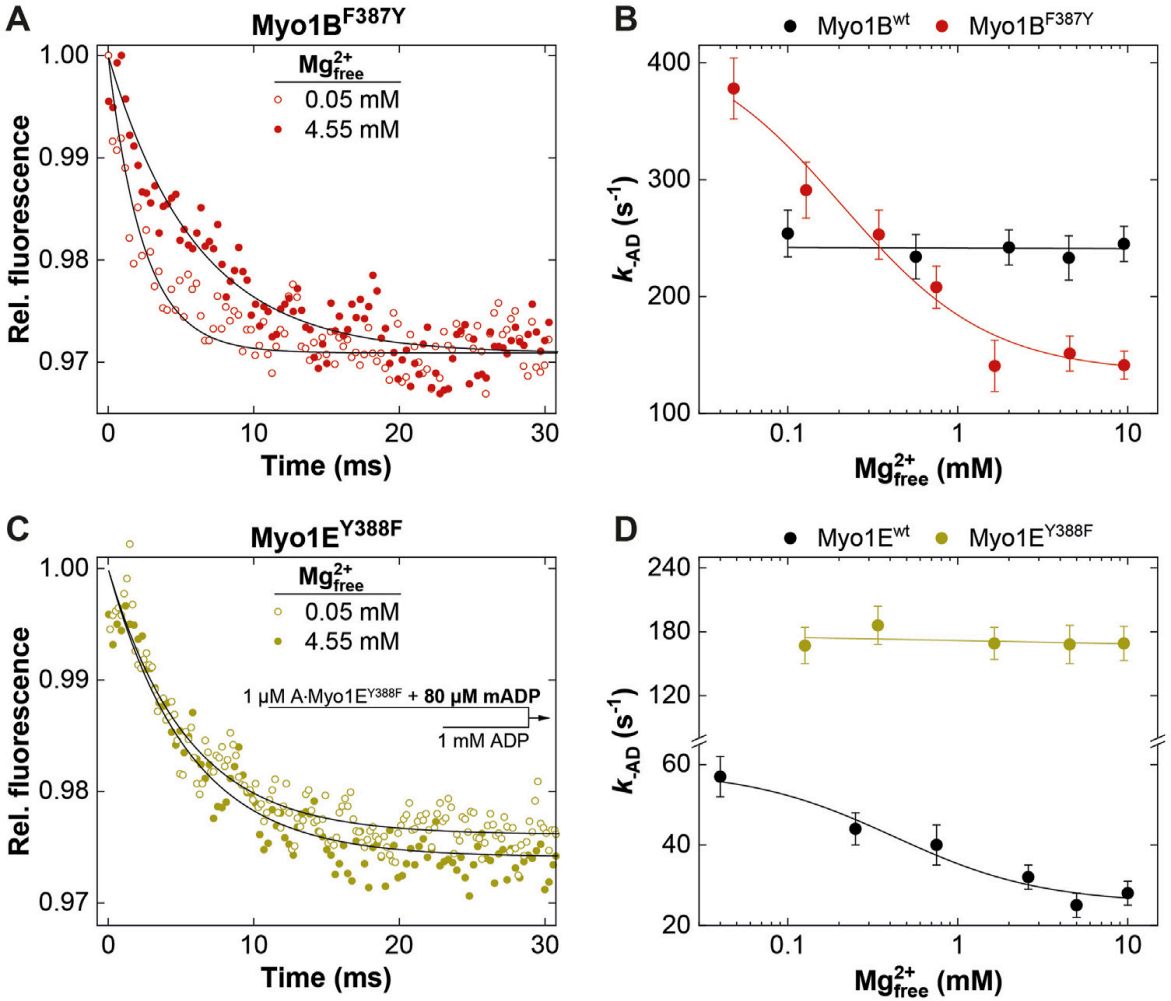
Effect of [Mg^2+^]_free_ on ADP release from actomyosin. Relative mantADP-fluorescence time traces as observed upon mixing 1 μM A∙Myo1B^F387Y^ **(A)** or 1 μM A∙Myo1E^Y388F^ **(C)** pre-equilibrated with 80 µM mADP at 0.05 mM and 4.55 mM free Mg^2+^-concentrations, with excess ADP. Rate of ADP release (***k***_**-AD**_) from A∙Myo1B^wt^ and A∙Myo1B^F388Y^ **(B)** and related Myo1E constructs **(D)** as a function of the free Mg^2+^-concentration as obtained from single exponential fits to the mantADP-fluorescence time traces.

First, we studied nucleotide interactions according to Figure 2 applying the kinetic models as described (Geeves et al., 1984; Taylor, 1991; Franz et al., 2021). Nucleotide binding in the absence of actin was mainly unaffected by the mutations (Table 2). However, both mutants displayed accelerated ADP release rates (*k*_-D_, Figure 3) and two to three-fold weaker affinities for ADP than the corresponding wild-types (K_D_; Table 2). The Y-to-F mutation in Myo1E^Y388F^ accelerated both rates of the two-step ADP release (Dürrwang et al., 2006) by up to four-fold, whereas mutant and wild-type Myo1B displayed single-step ADP release kinetics that differed by approx. two-fold. We interpret the accelerated ADP release as the major contributor of the reduced ADP affinity. In the presence of actin, equilibrium and rate constants of the interaction with ATP, defined by **K**_1_ and ***k***_**+2**_ (Figure 2), were differently affected in the mutants (Table 2). Myo1B^F387Y^ displayed five-fold slower ATP-binding (**K**_1_ ***k***_**+2**_) and three-fold lower ATP affinity of the A·M state. Myo1E^Y388F^ showed wild-type like ATP-binding behavior and a higher ATP-affinity of the A·M state than the wild-type. The tyrosine appears to disturb high affinity ATP binding by affecting the isomerization of the A·M·T state to the A-M*·T state. Pronounced changes were also observed for the ADP affinity of the A·M state (**K**_**AD**_) determined from the inhibition of the ATP-induced dissociation of the actomyosin complex with increasing ADP concentrations (Figure 4). Here, it is important to highlight the monophasic (Figures 4A, D) vs. biphasic actomyosin dissociation kinetics (Figures 4B, C) between wild-type and mutants. Monophasic dissociation kinetics are typical for myosins with a low affinity for ADP (Tsiavaliaris et al., 2002). A biphasic actomyosin dissociation reaction, in which the amplitude of the fast phase decreases and the amplitude of the slow phase increases with excess ADP, is indicative for a highly favorable A·M·D state, where ADP and ATP compete for the same binding site (Batra et al., 1999; Dürrwang et al., 2006). For both single and biphasic dissociation reactions, the ADP dependence of the rate (Figures 4A, D) and amplitude (Figures 4B, C) could be described by hyperbolic functions yielding **K_AD_** values listed in Table 2. Myo1B^F387Y^ displayed a five-fold higher affinity for ADP than Myo1B^wt^, whereas the ADP affinity of the actin-bound state of Myo1E^Y388F^ (A·M·D) was more than 6-fold decreased compared to wild-type. Thus, the F-to-Y substitution in Myo1B^F387Y^ appears to strengthen ADP binding and concomitantly prolong the strongly actin-bound states of the motor, while the Y-to-F substitution in Myo1E^Y388F^ induces the opposite effects. This altered ADP affinity is also reflected in the ADP release kinetics (***k***_**-AD**_, Figure 5), which were almost 2-fold decelerated for Myo1B^F387Y^ and more than six-fold accelerated for Myo1E^Y388F^ compared to the wild-types (Table 2). The mutations also displayed an inverse effect of the actin affinity of the ADP-bound states. Myo1B^F387Y^ displayed a more than 30-fold higher actin affinity in complex with ADP (**K**_**DA**_) compared to the wild-type, whereas Myo1E^Y388F^ showed a more than six-fold reduced actin affinity (Table 3). These prominent changes suggest an important role of the tyrosine for the mechano-chemical coupling mechanism of the class-1 myosins, which we investigated further.

**TABLE 2:** Rate and equilibrium constants of nucleotide interactions in the presence and absence of actin.

Nucleotide	Constant	Myo1B^wt^ ^c^	Myo1B^F387Y^	Myo1E^wt^ ^c^	Myo1E^Y388F^
Nucleotide binding to myosin					
ATP	K_1_ *k*_+2_ (µM^-1^s^-1^)	1.97 ± 0.01	weak signal	0.96 ± 0.03	1.1 ± 0.02
	*k*_+2_ (s^-1^)	>1,000	n.a.	900 ± 215	750 ± 190
	1/K_1_	350 ± 25	n.a.	940 ± 45	680 ± 32
mantATP	K_1_ *k*_+2_ (µM^-1^s^-1^)	1.3 ± 0.02	1.54 ± 0.1	0.91 ± 0.01	1.08 ± 0.1
ADP	*k*_+D_ (µM^-1^s^-1^)^a^	1.2 ± 0.3	1.4 ± 0.3	0.34 ± 0.02	0.69 ± 0.1
	*k*_-D1_ (s^-1^)	0.7 ± 0.003	1.26 ± 0.06	6.39 ± 0.51^d^	26.4 ± 1.9
	*k*_-D2_ (s^-1^)	n.a.	n.a.	1.77 ± 0.24^d^	5.17 ± 0.52
	K_D_ (µM)	0.4 ± 0.10	1.13 ± 0.23	7.1 ± 0.4^d^	25.8 ± 2.1
Nucleotide binding to actomyosin					
ATP	**K**_**1**_ ***k***_**+2**_ (µM^-1^s^-1^)	1.11 ± 0.03	0.22 ± 0.01	0.4 ± 0.01	0.41 ± 0.04
	***k***_**+2**_ (s^-1^)	>1,000	790 ± 62	750 ± 54	393 ± 18
	**1/K**_**1**_ (µM)	>900	3050 ± 276	1875 ± 152	975 ± 101
ADP	***k***_**+AD**_ (µM^-1^s^-1^)	4.95 ± 0.5	15 ± 2	2.5 ± 0.5	0.97 ± 0.1
	***k***_**-AD**_ (s^-1^)	233 ± 20^d^	150 ± 13	28 ± 4	180 ± 18
	**K**_**AD**_ (µM)	47 ± 5^d^	10 ± 1	12 ± 2	81 ± 17
	**K**_**AD**_ **/**K_D_	118 ± 32	9 ± 2	1.7 ± 0.3	3.1 ± 0.7
	***k***_**-** **AD**_ **/** *k*_-D1_	333 ± 29	115 ± 11	4.4 ± 0.7	6.8 ± 0.84
	***k***_**-** **AD**_ **/** *k*_-D1_	n.a.	n.a.	15.8 ± 3.1	34.8 ± 4.9
	K_i_ [Mg^2+^]_free_	n.a.	400 ± 45	800 ± 145	n.a.
Duty ratio^b^		0.01 ± 0.002	>0.01	0.55 ± 0.13	0.07 ± 0.01

^a^ *k*_+D_ = *k*_-D_/K_D_.

^b^Calculated from Eq. 2.

^c^ Depicted from Dürrwang et al., 2006 or Tsiavaliaris et al., 2008.

^d^ data from this study.

**TABLE 3:** Rate and equilibrium constants of actin interactions.

Constant	Myo1B^wt^*	Myo1B^F387Y^	Myo1E^wt^*	Myo1E^Y388F^
Myosin binding to actin				
***k***_**+A**_ (µM^-1^s^-1^)	1.1 ± 0.2	0.9 ± 0.08	2.9 ± 0.2	4.2 ± 0.3
***k***_**-A**_ (s^-1^)	0.005 ± 0.001	0.002 ± 0.0005	0.0015 ± 0.0003	0.0015 ± 0.0004
**K**_**A**_ (nM)	4.5 ± 1.0	2.2 ± 0.6	0.5 ± 0.1	0.4 ± 0.1
Myosin binding to actin in the presence of ADP				
***k***_**+DA**_ (µM^-1^s^-1^)	0.03 ± 0.004^a^	0.29 ± 0.04^a^	6.0 ± 0.8^a^	4.1 ± 0.6^a^
***k***_**-DA**_ (s^-1^)	0.017 ± 0.002	0.005 ± 0.001	0.005 ± 0.001	0.0007 ± 0.0001
**K**_**DA**_ (nM)	567 ± 100	17 ± 4	1.0 ± 0.2	6.1 ± 1.3 0.2
**K**_**DA**_ **/K**_**A**_	126 ± 36	8 ± 3	2.0 ± 0.6	15.3 ± 5.0

^a^ Calculated.

### 3.3 Switch-2 mutations alter ADP release kinetics in a Mg^2+^-controlled manner

It is well known that ***k***_**-AD**_ is the major determining parameter of the duty ratio (Mikhailenko et al., 2008; Taft et al., 2008; Diensthuber et al., 2015). In case that ***k***_**-AD**_ is of similar order as ***k***_**cat**_, the ADP release can limit the overall ATPase, which is a discernible characteristic of processive motors and motors made for tension, such as *Mm*myosin-5a, *Dd*myosin-5b, *Dd*myosin-1E, *Hs*myosin-7a, or *Nt*myosin-11 (Dürrwang et al., 2006; Watanabe et al., 2006; Sakamoto et al., 2008; Taft et al., 2008; Diensthuber et al., 2015). Contrary, the rate-limiting step of low duty ratio myosins is commonly the actin-accelerated P_i_-release, which precedes the fast dissociation of Mg^2+^ADP (Takagi et al., 2004). Previously, we and others have reported the existence of an equilibrium between magnesium free (A**·**M**·**D) and magnesium bound actomyosin-ADP (A**·**M**·**Mg^2+^D) states in high-duty ratio myosins (De La Cruz et al., 2000; Hannemann et al., 2005; Rosenfeld et al., 2005; Taft et al., 2008; Jacobs et al., 2011) through which product dissociation can occur either sequentially, where Mg^2+^ is released prior to ADP (Rosenfeld et al., 2005) or together with ADP (Chizhov et al., 2013). The preference for either path depends on free Mg^2+^-ions, which shift the equilibrium towards the simultaneous release of Mg^2+^ and ADP (Chizhov et al., 2013). Therefore, the fraction of time the myosin remains strongly attached to actin can be affected by free Mg^2+^-ions. For high duty ratio myosins we have shown that elevated, physiologically relevant free Mg^2+^ concentrations can inhibit the ADP release to such an extent that Mg^2+^ADP dissociation from the actin-bound states becomes the rate-limiting step, strongly influencing the motile properties and the duty ratio of these motors (Fujita-Becker et al., 2005; Dürrwang et al., 2006; Taft et al., 2008; Nagy et al., 2010; Diensthuber et al., 2015; Amrute-Nayak et al., 2019). The low duty ratio myosin Myo1B^wt^ (Tsiavaliaris et al., 2008) or skeletal class-2 myosin isoforms do not display such Mg^2+^-dependence of the ADP-release kinetics and motor activity (Taft et al., 2008). To examine whether the myosin isoform-specific sensitivity towards free Mg^2+^-ions was affected by the mutation, we measured the rate of ADP release from the actin-bound states using the fluorescent analogue mantADP. By displacing the bound mantADP with excess ADP in the presence of increasing concentrations of free Mg^2+^-ions at constant ionic strength (Figure 5), we obtained the rates of ADP release from single exponential fits of the fluorescence decays (Figures 5A, C). Interestingly, Myo1B^F387Y^ displayed ADP release rates (*k*_-AD_) that were dependent on free Mg^2+^, contrary to the wild-type, which did not show such a behavior (Figure 5B). The rates declined hyperbolically from initially 400 ± 25 s^-1^ (at 0.05 mM free Mg^2+^) to 120 ± 25 s^-1^ (at 10 mM free Mg^2+^) with an apparent inhibition constant K_i_^Mg^ of 0.45 ± 0.3 mM. The Myo1E constructs displayed exactly the opposite behavior: Myo1E^wt^ showed a Mg^2+^-dependence of ***k***_**-AD**_ as previously published (Dürrwang et al., 2006), whereas Myo1E^Y388F^ displayed ADP release rates that were independent of free Mg^2+^-ions (Figure 5D) yielding values comparable to those of Myo1B^wt^ (Figure 5B). All other experimentally determined kinetic parameters listed in the tables showed no or only minor changes. We therefore omitted graphical representation of the data. In summary, the tyrosine in switch-2 of class-1 myosins appears to play a determining role for Mg^2+^ADP release, which could also affect the motile properties and duty ratio of the myosins.

### 3.4 Switch-2 mutations inversely affect duty ratio and motor activity as a consequence of altered thermodynamic and kinetic coupling

**FIGURE 6:**
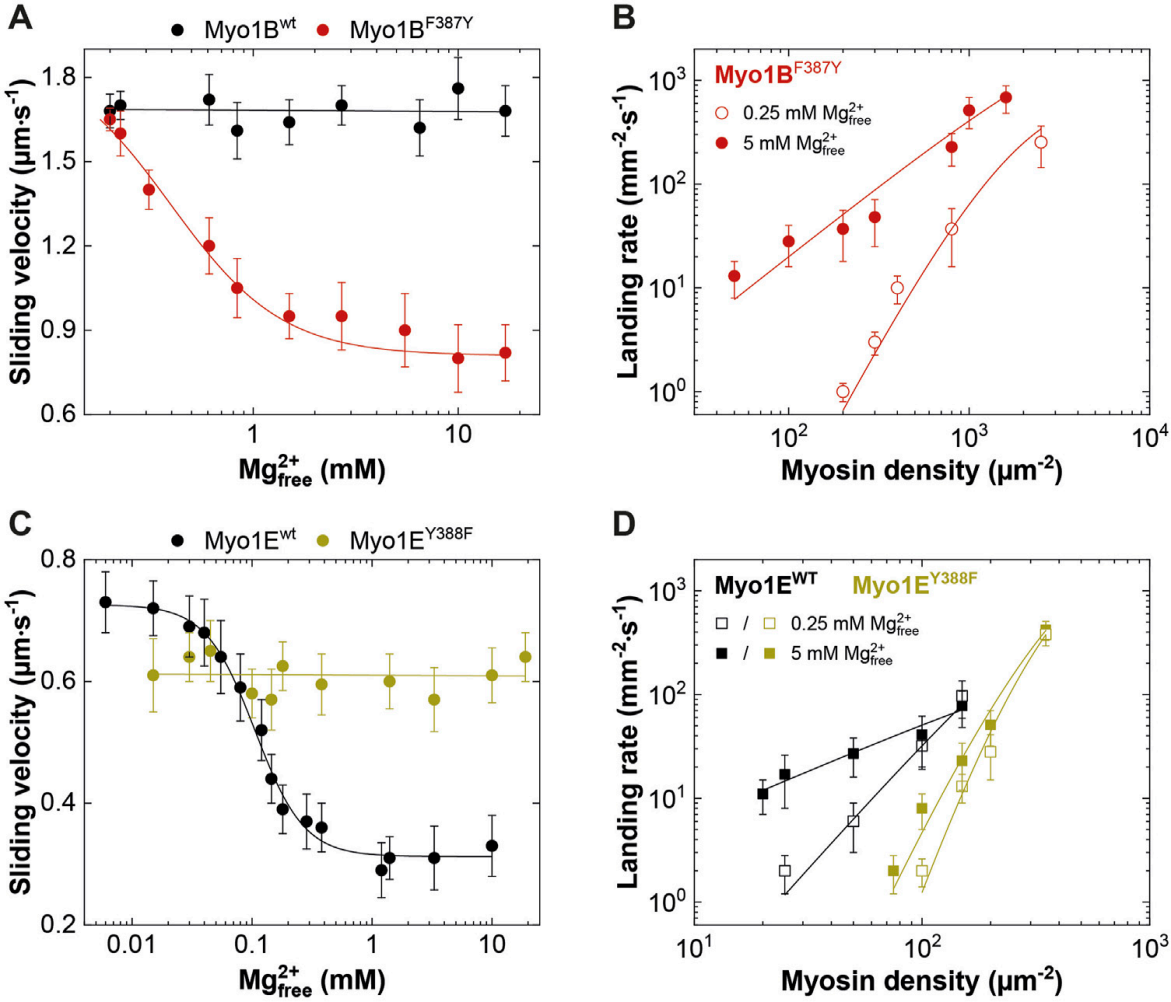
Effect of [Mg^2+^]_free_ on myosin motor activity and landing rate. **(A,C)** Average actin sliding velocities of wild-type and mutant myosins at increasing free Mg^2+^-concentrations. Myo1B^wt^ and Myo1E^Y388F^ display no Mg^2+^ sensitivity. **(B)** Landing rate as a function of Myo1B^F387Y^ motor density. **(D)** Landing rate as a function of Myo1E^wt^ and Myo1E^Y388F^ motor density. The landing assays were performed at 5 mM (filled symbols) and 0.25 mM (open symbols) free Mg^2+^ concentrations.

To investigate this, we generated motor domain constructs with artificial lever arms and performed *in vitro* motility assays as previously described (Taft et al., 2008). The experiments were performed under constant ionic strength and revealed an inhibitory effect of free Mg^2+^ on the actin sliding velocity of Myo1E^wt^ and Myo1B^F387Y^ but not on that of Myo1B^wt^ and Myo1E^Y388F^ (Figures 6A, C). We note that for Myo1E^wt^, the rate constant for ADP release (***k***_**-AD**_) at excess free Mg^2+^-concentrations is similar to ***k***_**cat**_ and thus the rate-limiting parameter of the ATPase. For the mutant Myo1E^Y388F^, the ADP release is not a rate limiting factor of the ATPase cycle, since it proceeds by more than one order of magnitude faster than the steady-state ATP turnover (***k***_**cat**_). This hints for a low duty ratio. This kinetic data reveal that the tyrosine is an important residue in switch-2 that controls Mg^2+^ADP release and potentially the weak-to-strong actin binding interactions. The thermodynamic coupling constants (**K**_**AD**_/K_D_; **K**_**DA**_/**K**_**A**_) and the kinetic coupling constant (***k*-**_**AD**_ **/** *k*_-D_) of nucleotide and actin binding are valuable parameters related to weak-to-strong actin binding transitions (Bloemink and Geeves, 2011). They report how effectively actin can displace ADP, providing predictions of the duty ratio of a myosin. High duty ratio myosins display low coupling constants that tend to approximate unity or acquire values below 1, since the displacement of ADP from myosin by actin is slowed down, often rate limiting the ATPase cycle, which contributes to prolonged population of the strongly actin bound states. Myo1E^wt^ displays typical coupling constants (**K**_**AD**_/**K**_**D**_ = 1.7 ± 0.3; **K**_**DA**_/**K**_**A**_ = 2.0 ± 0.6; ***k*-**_**AD**_ **/** *k*_-D1_ = 4.4 ± 0.7; ***k*-**_**AD**_ **/** *k*_-D2_ = 15.8 ± 3.1; Table 2, 3) of a high duty ratio motor, whereas the coupling constants of Myo1B^wt^ (**K**_**AD**_/**K**_**D**_ = 118 ± 32; **K**_**DA**_/**K**_**A**_ = 126 ± 36; ***k*-**_**AD**_ **/** *k*_-D_ = 333 ± 29; Table 2, 3) resemble those of fast motors with a low duty ratio characterized by an effective actin-stimulated ADP release (Bloemink and Geeves, 2011). Notably, the mutations almost reversed the coupling parameters. The Y-to-F substitution in Myo1E^Y388F^ led to an increase of the coupling constants by two- to seven-fold (**K**_**AD**_/**K**_**D**_ = 3.1 ± 0.7; **K**_**DA**_/**K**_**A**_ = 15.3 ± 5.0; ***k*-**_**AD**_ **/** *k*_-D1_ = 6.8 ± 0.8; ***k*-**_**AD**_ **/** *k*_-D2_ = 34.8 ± 4.9; Table 2, 3), which indicates a decrease of the duty ratio. The F-to-Y substitution in Myo1B^F387Y^ caused a decrease of all coupling constants (**K**_**AD**_/**K**_**D**_ = 9 ± 2; **K**_**DA**_/**K**_**A**_ = 8 ± 3; ***k*-**_**AD**_ **/** *k*_-D_ = 115 ± 11; Table 2, 3), indicating that the duty ratio is also affected. Using Eq. 2 (Watanabe et al., 2006; Ito et al., 2007), we calculated the duty ratios (Table 2) revealing for the mutant Myo1E^Y388F^ indeed a lower duty ratio (0.07 ± 0.01) compared to Myo1E^wt^ (0.55 ± 0.13). For Myo1B^wt^ we obtained a low duty ratio (0.01 ± 0.01), while the calculation of the duty ratio of Myo1B^F387Y^ predicts a value > 0.01.

Although the duty ratios calculated from the kinetic data correlate well with the coupling parameters, the values appear to be underestimated, particularly for the Myo1B constructs. This is due to the high uncertainty in determining ***k***_**cat**_. To experimentally validate the effect of the mutations on the duty ratio, we performed landing assays at two boundary conditions (0.25 mM and 5 mM free Mg^2+^ concentrations) to account for the Mg^2+^-effect observed in the kinetics and motility assays (Figure 5; Figures 6A, C). The number of motile landing events decreased with decreasing motor densities (Figures 6B, D). The dependency of the landing rates on motor density was best fit by Eq. 1 as described (Hancock and Howard, 1998). From the reciprocal of n, the duty ratio could be calculated (Sumiyoshi et al., 2007), revealing that the duty ratio of Myo1E^wt^ increased from 0.38 ± 0.11 at 0.25 mM free Mg^2+^ to 0.91 ± 0.07 at 5 mM free Mg^2+^, whereas the duty ratio of Myo1E^Y388F^ dropped to 0.14 ± 0.02 at 0.25 mM free Mg^2+^ and 0.2 ± 0.04 at 5 mM free Mg^2+^, respectively. This demonstrates the loss of the Mg^2+^-sensitivity of the motor (Table 4) consistent with Mg^2+^-insensitive ADP-release kinetics (Figure 5) and Mg^2+^-insensitive motor activity (Figures 6A, C). Myo1B^wt^ was significantly more difficult to handle in the landing assays. At densities below 5000 motor molecules µm^-2^, we observed almost no landing events, which speaks for a low duty ratio of the motor. Therefore, there are no data available for Myo1B^wt^. Myo1B^F387Y^ displayed landing events that were dependent on both, free Mg^2+^-ions and motor density, yielding duty ratios of 0.14 ± 0.02 at 0.25 mM Mg^2+^ and 0.5 ± 0.1 at 5 mM free Mg^2+^. The estimation of the duty ratio for Myo1B^F387Y^ should be taken with care, since the number of moving actin filaments at low motor densities became increasingly less. However, the data clearly demonstrate that the introduction of the tyrosine in Myo1B significantly increases the duty ratio of the myosin.

**TABLE 4:** Regulation of myosin motor activity and duty ratio.

Parameter	Myo1B^wt^ ^a^		Myo1B^F387Y^ ^a^		Myo1E^wt^ ^a^		Myo1E^Y388F^ ^a^	
*In vitro*motility assays								
v_max_ (µm s^-1^)	1.76 ± 0.11		1.68 ± 0.06		0.73 ± 0.05		0.64 ± 0.06	
v_min_ (µm s^-1^)	1.61 ± 0.09		0.82 ± 0.1		0.31 ± 0.05		0.58 ± 0.05	
K_i_ (mM)	n.a.		0.19 ± 0.03		0.13 ± 0.02		n.a.	
Hill coefficient (n)	n.a.		1.96 ± 0.17		2.11 ± 0.21		n.a.	
Landing assays								

	[Mg^2+^]_free_		[Mg^2+^]_free_		[Mg^2+^]_free_		[Mg^2+^]_free_	
	250 µM	5 mM	250 µM	5 mM	250 µM	5 mM	250 µM	5 mM
Landing rate order	n.a.		7.2 ± 1.5	2 ± 1	2.6 ± 0.8	1.1 ± 0.1	7 ± 1.1	5.1 ± 0.9
Duty ratio (n^−1^)	n.a.		0.14 ± 0.02	0.5 ± 0.1	0.38 ± 0.11	0.91 ± 0.07	0.14 ± 0.02	0.2 ± 0.04

^a^ Data of this study.

### 3.5 Switch-2 mutations influence Mg^2+^ADP release through altered interactions of active site elements

**FIGURE 7:**
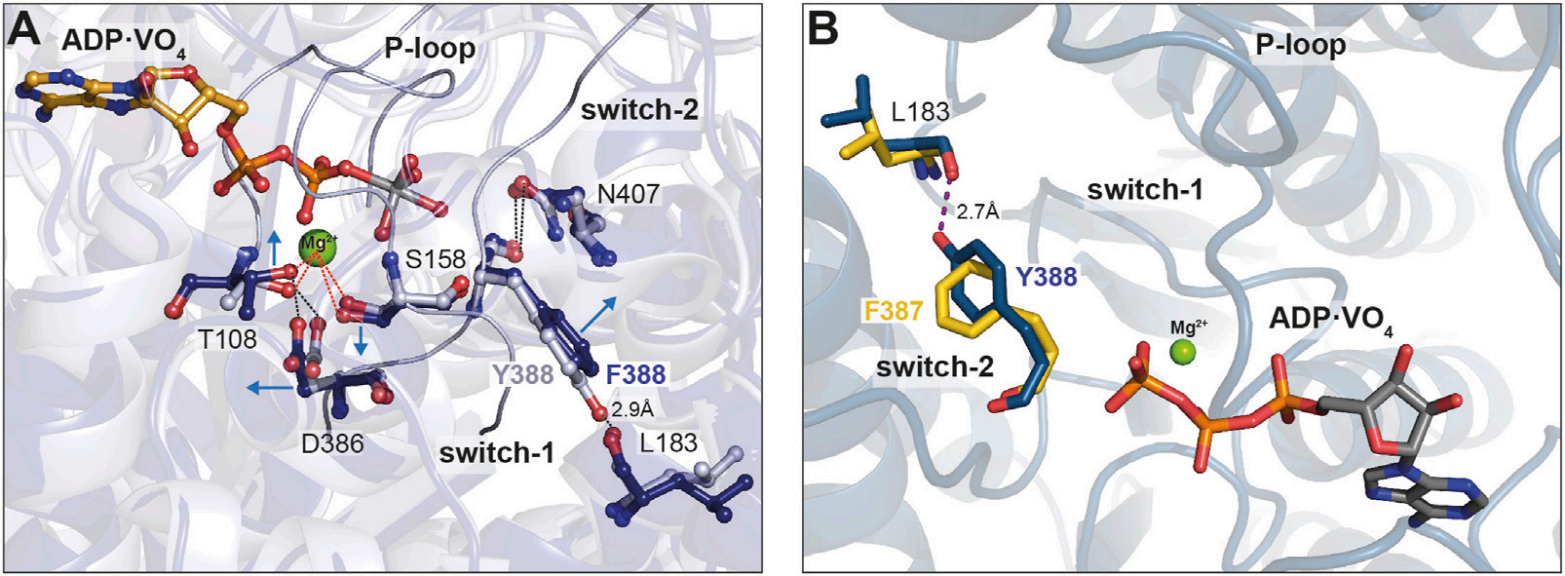
Close up view of the switch-2 region of the motor domain of Myo1B and Myo1E. **(A)** Superposition of structural models showing the influence of the Y-to-F mutation in Myo1E on Mg^2+^ and ADP coordination. The introduction of mutation Y388F prevents the formation of a hydrogen bond with L183, which stabilizes the nucleotide coordination sphere in the wild-type. The arrows indicate the positional shift of switch-2 residues induced by the mutation. The altered conformation of D386 (switch-2) strengthens the hydrogen bond with T108 (P-loop) and weakens the T108-mediated coordination of the Mg^2+^. **(B)** Superimposed structural models showing the influence of the F-to-Y substitution in Myo1B. The Tyr in Myo1B^F387Y^ can adopt a conformation that allows the formation of a hydrogen bond with residue L183, in a similar manner as in Myo1E^wt^. The hydrogen bond is assumed to stabilize the conformation of switch-2 enhancing the coordination capacity for Mg^2+^ and nucleotide. The images were created using the “Pymol” program.

Finally, we used the available X-ray structure of Myo1E (pdb: 1LKX) (Kollmar et al., 2002) and generated energy minimized homology models of Myo1E^Y388F^, which we applied to molecular dynamics simulations to obtain insights into potential conformational changes induced by the mutation (Figure 7). The superimposed structures in complex with ADP-VO_4_ show Y388 in switch-2 of wild-type Myo1E^wt^ to form a 2.9 Å hydrogen bond with the β7-sheet residue L183 of the transducer (Figure 7A). This conformation appears to stabilize the Mg^2+^-ion in the binding pocket of the wild-type myosin. The structural model of Myo1E^Y388F^ predicts the loss of this hydrogen bond (Figure 7B). As a consequence switch-2 is slightly shifted, which enables residue D386 to strengthen the hydrogen bond with P-loop residue T108. This in turn weakens the T108-mediated coordination of the Mg^2+^-ion. This conformational shift of switch-2 appears to additionally affect the Mg^2+^-coordination mediated via switch-1 residue S158. Consequently, these small conformational rearrangements of switch-2 appear to disrupt the coordination sphere of Mg^2+^, which is apparently associated with an accelerated Mg^2+^-ADP release and the inability of the Myo1E^Y388F^ to sense free Mg^2+^-ions. For Myo1B we generated energy-minimized homology models (Figure 7B) to show that a hydrogen bond between Y387 in the mutant and L183 can be formed like in Myo1E^wt^, which is absent in the wild-type (Figure 7B, blue residues). This tyrosine-mediated hydrogen bond may be critical for stabilizing Mg^2+^ and ADP, which could explain the Mg^2+^-dependent suppression of the ADP-release from acto·Myo1B^F387Y^ and the prolonged strongly actin-bound states responsible for the higher duty ratio. These structural considerations are supported by X-ray and cryoEM structures of Myo5, revealing that the tyrosine in switch-2 is important for stabilizing the rigor conformation in high duty ratio motors via the interaction with leucine in the ß7 sheet (Coureux et al., 2004; Pospich et al., 2021). Mechanistically, the effect of free Mg^2+^ can be interpreted as previously described for Myo5b (Rosenfeld et al., 2005; Chizhov et al., 2013). The dissociation of the Mg^2+^ can occur prior or in complex with ADP. Thus, increased concentrations of free Mg^2+^-ions can slow down the release of the nucleotide. Our structural models attribute this to the altered switch-2 conformation induced by the mutations affecting the ADP affinity of the actomyosin complex. The tyrosine stabilizes the Mg^2+^·ADP state, while the phenylalanine causes accelerated release rates. However, the F-to-Y mutation in MyoB^F387Y^ did not alter the ADP dissociation from the acto-MyoB^F387Y^ complex to rate-limit the overall ATPase cycle time, as in the case of MyoBF387Y. Obviously, other structural elements including switch-1, loop-1, W-helix, and loop-2, all of which have been related to product release (Bobkov et al., 1996; Sweeney et al., 1998; Murphy and Spudich, 1999; Clark et al., 2005; Bloemink et al., 2014; Franz et al., 2021), contribute additionally in fine-tuning the ADP release kinetics. With respect to switch-2, our data suggest the tyrosine in class-1 myosins suppresses the actin-induced acceleration of ADP release by strengthening the coordination of Mg^2+^ and ADP through the stabilization of the conformation of the surrounding active site elements.

### 3.6 Conclusions

The presence of the tyrosine in the switch-2 consensus sequence of myosins is not strictly indicative for a high duty ratio. For example, mammalian class-I myosins contain a tyrosine and display under unloaded conditions characteristics of a low duty ratio motor (Greenberg and Ostap, 2013), whereas under tension (Greenberg et al., 2012; Pyrpassopoulos et al., 2016) and/or Ca^2+^-dependent binding of calmodulin to the neck region (Lewis et al., 2012), they exhibit altered rates and equilibrium constants of the transitions in the ATPase cycle, which can increase the duty ratio (Laakso et al., 2008). Interestingly, the class-VI myosin members are high duty ratio motors, although they possess an alanine at the Y388-equivalent position (De la Cruz et al., 2001). Furthermore, the exchange of the tyrosine in switch-2 of mammalian myosin-5a by an alanine resulted in an increased processive behaviour of the motor and strengthened the free Mg^2+^-dependent suppression of ADP release from actomyosin, however, at reduced speed (Nagy et al., 2010). These findings suggest that natural variations of structural elements besides switch-2, including those mediating actin interactions, such as loop-2, loop-4, the CM-loop, activation loop, helix-loop-helix (Goodson et al., 1999; Onishi et al., 2006; Diensthuber et al., 2015) have a dominant role in the regulation of the duty ratio, since they couple actin-interactions to product release as exemplary shown for class-V and class-I (Yengo and Sweeney, 2004; Lieto-Trivedi et al., 2007).

Here, we provide kinetic and molecular insights into the product release mechanisms of two functionally distinct class-1 myosins and reveal that switch-2 dictates the duty ratio of the motors and their ability to perform rapid movement, processive motion, or gating function. The Mg^2+^-sensitivity of the class-1 myosins appears to be an important feature in cellular processes that require a switching between fast contractility and slow tension bearing, such as during endocytosis, where the myosins act as motorized cross-linkers between the membrane and actin cytoskeleton systems to provide the contractile forces to accomplish actin and membrane remodelling (Manenschijn et al., 2019). Additionally, class-1 myosins can act as tension-sensitive tethers or even transporters (McIntosh and Ostap, 2016). Thus, it would be interesting to analyse how switch-2 in conjunction with other structural elements implicated in regulating Mg^2+^ADP release define the duty ratio of individual myosin-1 isoforms to perform distinct and multiple types of molecular functions.

## Data Availability

The original contributions presented in the study are included in the article/supplementary material, further inquiries can be directed to the corresponding author.
